# Mortality and cancer in relation to ABO blood group phenotypes in the Golestan Cohort Study

**DOI:** 10.1186/s12916-014-0237-8

**Published:** 2015-01-15

**Authors:** Arash Etemadi, Farin Kamangar, Farhad Islami, Hossein Poustchi, Akram Pourshams, Paul Brennan, Paolo Boffetta, Reza Malekzadeh, Sanford M Dawsey, Christian C Abnet, Ashkan Emadi

**Affiliations:** Digestive Oncology Research Center, Digestive Disease Research Institute, Tehran University of Medical Sciences, Tehran, Iran; Division of Cancer Epidemiology and Genetics, National Cancer Institute, 9609 Medical Center Dr, Bethesda, MD 20859 USA; Department of Public Health Analysis, School of Community Health and Policy, Morgan State University, Baltimore, MD USA; Surveillance and Health Services Research, American Cancer Society, Atlanta, GA USA; Liver and Pancreatobiliary Research Center, Digestive Disease Research Institute, Tehran University of Medical Sciences, Tehran, Iran; International Agency for Research on Cancer, Lyon, France; Institute for Translational Epidemiology and Tisch Cancer Institute, Icahn School of Medicine at Mount Sinai, New York, NY USA; Greenebaum Cancer Center, University of Maryland, Baltimore, MD USA

**Keywords:** Blood group, ABO, Rh, Mortality, Cancer, Cardiovascular disease

## Abstract

**Background:**

A few studies have shown an association between blood group alleles and vascular disease, including atherosclerosis, which is thought to be due to the higher level of von Willebrand factor in these individuals and the association of blood group locus variants with plasma lipid levels. No large population-based study has explored this association with overall and cause-specific mortality.

**Methods:**

We aimed to study the association between ABO blood groups and overall and cause-specific mortality in the Golestan Cohort Study. In this cohort, 50,045 people 40- to 70-years old were recruited between 2004 and 2008, and followed annually to capture all incident cancers and deaths due to any cause. We used Cox regression models adjusted for age, sex, smoking, socioeconomic status, ethnicity, place of residence, education and opium use.

**Results:**

During a total of 346,708 person-years of follow-up (mean duration 6.9 years), 3,623 cohort participants died. Non-O blood groups were associated with significantly increased total mortality (hazard ratio (HR) = 1.09; 95% confidence interval (CI): 1.01 to 1.17) and cardiovascular disease mortality (HR = 1.15; 95% CI: 1.03 to 1.27). Blood group was not significantly associated with overall cancer mortality, but people with group A, group B, and all non-O blood groups combined had increased risk of incident gastric cancer. In a subgroup of cohort participants, we also showed higher plasma total cholesterol and low-density lipoprotein (LDL) in those with blood group A.

**Conclusions:**

Non-O blood groups have an increased mortality, particularly due to cardiovascular diseases, which may be due to the effect of blood group alleles on blood biochemistry or their effect on von Willebrand factor and factor VIII levels.

Please see related commentary 10.1186/s12916-014-0250-y.

## Background

E.B. Ford, the renowned geneticist, was quoted in 1945 as saying, ‘It is reasonable to conclude, from what we know of polymorphisms, that individuals belonging to the different blood groups are not equally viable…’ [1]. Although blood group antigens have been widely recognized because of the complications they produce in transfusion medicine [2], their conservation through evolution and their presence on many cells in the human body [1] suggest they are also critical to human physiology. However, the only documented roles, so far, include susceptibility to certain infections such as *Plasmodium falciparum* [3] and *Helicobacter pylori* [4], and the level and structure of the von Willebrand Factor (vWF)-FVIII complex in blood [2].

The ABO(H) blood group system was the first genetic polymorphism discovered in humans [5]. So, it is not surprising that it has been studied in the context of many chronic diseases. Many vascular disorders (especially venous thromboembolism and atherosclerotic disease) have been linked to non-O blood group status [6]. This association is thought to be mainly due to the higher level of factor VIII and vWF in these individuals, and to some extent the association of blood group locus variants with plasma lipid levels, especially cholesterol [7]. Higher levels of factor VIII and vWF lead to increased thrombotic tendency [8], and plasma cholesterol is a known risk factor for atherosclerosis. ABO blood groups have also been extensively studied in association with cancer. Some of the most consistent associations observed so far include the associations between non-O blood groups and pancreatic cancer (which was also confirmed in a genome-wide association study (GWAS) [9]), and between the A blood group and gastric cancer [10] and atrophic gastritis [11].

Despite their discovery in 1900, the critical role of ABO blood groups in transfusion medicine, and their apparent link to multiple diseases, the association of blood groups with mortality in the general population has not been evaluated in a large prospective study [6]. Therefore, we decided to examine the hypothesis that blood groups are associated with overall and cause-specific mortality, using the data from the large prospective Golestan Cohort Study.

## Methods

Details of the Golestan Cohort Study (GCS) have been published before [12]. This study is a population-based cohort in northeastern Iran which has followed 50,045 people above the age of 40 since 2004. At cohort recruitment, between the years 2004 and 2008, all participants were interviewed by trained cohort staff and underwent blood group determination. ABO blood group and Rh could not be determined for four and two individuals, respectively, who were excluded from analyses.

The GCS was approved by the Institutional Review Boards of the Digestive Disease Research Center (DDRC), the US National Cancer Institute (NCI), and the International Agency for Research on Cancer (IARC), and all participants gave written informed consent before enrollment.

Details of the GCS follow-up procedures have been published before [13]. Annual follow-up has had a 99% success rate so far. In these follow-up contacts, any case that is suspicious for cancer is evaluated and documented, and the records are complemented by linkage to local and national registries. Any reported death is also followed by a visit from a physician who completes a verbal autopsy questionnaire, validated for this population [13], by interviewing the closest relative of the deceased. At the same time, death certificates and all available medical documents are collected. The cause of death is classified according to the International Classification of Diseases, 10th revision (ICD-10) codes. For this analysis, causes of death were categorized as medical or external (that is, accidents, intoxication, suicide or other types of injury). Medical causes of death were further divided into cardiovascular disease (ischemic heart disease (ICD-10 codes I20-I25), cerebrovascular disease (I60-I69), and other diseases of the circulatory system); death due to cancer (ICD-10 codes C00-C97); and death due to other medical causes. Follow-up for this analysis continued until the subject was lost to follow-up, death occurred, or 28 February 2014, whichever came first.

In a random subgroup of the original cohort (n = 11,418), a second round of risk factor assessment and blood biochemistry tests was done four to five years after the initial enrollment. These results were used to analyze the association of blood groups with cardiovascular risk factors, including plasma lipids, blood glucose, blood pressure and anthropometric measurements.

We used Cox proportional hazards models, with age as the time variable, to estimate unadjusted and adjusted hazard ratios (HRs) and 95% confidence intervals (CIs) for mortality and cancer incidence, in relation to blood groups. Participants were left-censored at the age of enrollment, and all models (crude or adjusted) were adjusted for age at cohort baseline. The adjusted models also included potential confounders (sex, ethnicity, place of residence (urban or rural), education, quartiles of smoking in pack-years, opium use and an index of socioeconomic status [14]). These variables were selected because they have been shown to affect mortality in general or in this population [15]. Models for cancer incidence were adjusted for the same variables. The cancers used as outcomes were those having strong *a priori* associations with blood groups (gastric and pancreatic cancer) and the most common cancer in this population (esophageal squamous cell carcinoma). The follow up for these models continued until the loss to follow-up, death, cancer diagnosis, or 28 February 2014, whichever came first.

Population attributable fractions (AF_p_) were calculated using Levin’s formula [16]:

$$ A{F}_p=\frac{\left\{RR-1\right\}\times P{F}_1}{1+\left\{RR-1\right\}P{F}_1} $$

where PF_1_ is the proportion of the population in any given blood group, and RR is the relative risk of the outcome in that blood group compared with the reference risk (group O).

All statistical tests were two-sided and a *P* value of 0.05 or smaller was considered significant.

## Results

The most common blood group phenotype in this population was A (33.4%) followed by O (29.9%), and 93.5% were Rh positive. Table 1 shows the characteristics of the population across different blood groups. During a total of 346,708 person-years of follow-up (mean ± SD: 6.9 ± 1.5 years) through February 2014, 3,623 cohort participants died. The most common cause of death was cardiovascular disease (n = 1,879, 51.9%), followed by cancer (n = 775, 21.4%).

**Table 1:** **Baseline characteristics of Golestan Cohort Study participants by blood group phenotypes**

**Variable**		**O (number = 14,971)**	**A (number = 16,705)**	**B (number = 13,555)**	**AB (number = 4,810)**	**Rh + (number = 46,805)**	**Rh-(number = 3,238)**
**Sex**	female	8738 (58.4)	9555 (57.2)	7760 (57.2)	2757 (57.3)	26974 (57.6)	1837 (56.7)
	male	6233 (41.6)	7150 (42.8)	5795 (42.8)	2053 (42.7)	19831 (42.4)	1401 (43.3)
**Age** ^**a**^		52.1 (9)	52.1 (9)	52.2 (9.1)	52.1 (9)	52.1 (9.0)	52.2 (8.9)
**Ethnicity**	Turkmen	10836 (72.4)	12677 (75.9)	10033 (74)	3705 (77)	35060 (74.9)	2192 (67.7)
	non-Turkmen	4135 (27.6)	4028 (24.1)	3522 (26)	1105 (23)	11745 (25.1)	1046 (32.3)
**Residence**	urban	3022 (20.2)	3257 (19.5)	2794 (20.6)	955 (19.9)	9247 (19.8)	783 (24.2)
	rural	11949 (79.8)	13448 (80.5)	10761 (79.4)	3855 (80.1)	37558 (80.2)	2455 (75.8)
**Education**	none	10388 (69.4)	11785 (70.5)	9514 (70.2)	3431 (71.3)	32919 (70.3)	2199 (67.9)
	Up to 8 years	3246 (21.7)	3547 (21.2)	2910 (21.5)	1004 (20.9)	9985 (21.3)	723 (22.3)
	High school	993 (6.6)	1019 (6.1)	855 (6.3)	288 (6)	2929 (6.3)	226 (7)
	University	344 (2.3)	354 (2.1)	276 (2)	87 (1.8)	972 (2.1)	90 (2.8)
**Smoking pack-year**	Non-smoker	11751 (78.5)	13149 (78.7)	10581 (78.1)	3753 (78)	36685 (78.4)	2551 (78.8)
	Q1 (≤3)	858 (5.7)	934 (5.6)	783 (5.8)	272 (5.7)	2682 (5.7)	165 (5.1)
	Q2 (3 to 10)	772 (5.2)	880 (5.3)	695 (5.1)	233 (4.8)	2406 (5.1)	174 (5.4)
	Q3( 10 to 24)	820 (5.5)	900 (5.4)	757 (5.6)	289 (6)	2581 (5.5)	185 (5.7)
	Q4 (>24)	770 (5.1)	842 (5)	739 (5.5)	263 (5.5)	2451 (5.2)	163 (5)
**Opium use**		2535 (16.9)	2854 (17.1)	2306 (17)	804 (16.7)	7977 (17)	523 (16.2)
**Socioeconomic status**	Low	4065 (27.2)	4703 (28.2)	3803 (28.1)	1366 (28.4)	13054 (27.9)	884 (27.3)
	Low-middle	3268 (21.8)	3699 (22.1)	3092 (22.8)	1085 (22.6)	10464 (22.4)	680 (21)
	High-middle	3740 (25)	4286 (25.7)	3392 (25)	1167 (24.3)	11792 (25.2)	793 (24.5)
	High	3898 (26)	4017 (24)	3268 (24.1)	1192 (24.8)	11495 (24.6)	881 (27.2)
**Medical history at baseline**	CVD	959 (6.4)	1007 (6)	803 (5.9)	282 (5.9)	2834 (6.1)	217 (6.7)
	Hypertension	2968 (19.8)	3284 (19.7)	2726 (20.1)	896 (18.6)	9264 (19.8)	611 (18.9)
	DM	999 (6.7)	1178 (7.1)	952 (7)	325 (6.8)	3224 (6.9)	230 (7.1)
	Cancer	52 (0.4)	56 (0.3)	34 (0.3)	17 (0.4)	149 (0.3)	10 (0.3)

^a^Numbers show frequencies (percentage) except for age which is mean (SD). CVD, cardiovascular disease; DM, diabetes mellitus.

For mortality analyses, 209 deaths due to external causes were excluded, and only medical causes of death were considered. As Table 2 shows, non-O blood groups were associated with a significantly increased total mortality (HR = 1.09; 95% CI: 1.01 to 1.17), which was most pronounced for cardiovascular disease mortality (HR = 1.15; 95% CI: 1.03 to 1.27). In this population, 5.9% of total mortality due to medical causes, and 8.9% of those due to cardiovascular disease could be attributed to having non-O blood group. Among the different non-O blood groups, both groups A and B were individually associated with both higher overall and cardiovascular mortality, compared with group O. Cancer mortality was, to some extent, more frequent in individuals with non-O blood group alleles, although this difference was not statistically significant.

**Table 2:** **Overall and cause-specific mortality by blood group in the Golestan Cohort Study**

**Blood type**	**number (person-years)**	**deaths (MR)**	**Overall mortality due to medical causes** ^**a**^		**CVD mortality (number = 1,879)**	**Cancer mortality (number = 775)**	**Other medical causes (numner = 760)**
			**crude HR (95% CI)**	**adjusted HR** ^**b**^ **(95% CI)**	**adjusted HR (95% CI)**	**adjusted HR (95% CI)**	**adjusted HR (95% CI)**
O	14,971 (103,756)	1,007 (971)	1	1	1	1	1
A	16,705 (115,764)	1,223 (1,056)	1.11 (1.02,1.21)*	1.08 (1.00,1.18)*	1.12 (1.00,1.26)*	1.05 (0.87,1.25)	1.03 (0.86,1.23)
B	13,555 (93,740)	1,036 (1,105)	1.12 (1.03,1.23)*	1.09 (1.00,1.20)*	1.15 (1.02,1.30)*	1.14 (0.95,1.38)	0.91 (0.75,1.10)
AB	4,810 (33,434)	357 (1,068)	1.10 (0.97,1.25)	1.07 (0.95,1.22)	1.16 (0.98,1.37)	1.03 (0.79,1.35)	0.92 (0.70, 1.21)
Non-O	35,070 (242,966)	2,616 (1,077)	1.11 (1.03,1.20)*	1.09 (1.01,1.17)*	1.15 (1.03,1.27)**	1.08 (0.92,1.27)	0.97 (0.83,1.13)
Rh+	46,803 (324,155)	3,381 (1,043)	1	1	1	1	1
Rh-	3,238 (22,553)	242 (1,073)	1.04 (0.91,1.19)	1.06 (0.93,1.21)	1.04 (0.87,1.25)	0.88 (0.64,1.19)	1.30 (0.99,1.69)

^a^Excluding deaths due to accidents, intoxication, suicide or other types of injury; ^b^Cox regression models adjusted for age, smoking, socioeconomic status, ethnicity, residence, education, and opium use. *P <0.05, **P <0.01, CI, confidence ratio; CVD, cardiovascular disease; HR: hazard ratio; MR: mortality rate per 100,000 person-years.

People with a non-O blood group had an increased risk of gastric cancer (HR = 1.55; 95% CI: 1.09 to 2.21). The significantly increased risk was seen in both blood group A (HR = 1.57; 95% CI: 1.06 to 2.32) and B (HR = 1.59; 95% CI: 1.06 to 2.39). Other cancers were not associated with blood group phenotype (Table 3).

**Table 3:** **Incidence of three main cancer types by blood group in the Golestan Cohort Study**

**Blood type**	**Gastric cancer**		**ESCC**		**Pancreatic cancer**	
	**Number of cases**	**adjusted HR** ^**a**^ **(95% CI)**	**Number of cases**	**adjusted HR (95% CI)**	**Number of cases**	**adjusted HR (95% CI)**
O	39	1	69	1	20	1
A	73	1.57 (1.06,2.32)*	67	0.82 (0.58,1.15)	21	0.91 (0.49,1.68)
B	58	1.59 (1.06,2.39)*	62	0.94 (0.67,1.32)	13	0.70 (0.35,1.42)
AB	18	1.35 (0.77,2.36)	18	0.76 (0.45,1.27)	5	0.75 (0.28,2.00)
Non-O	149	1.55 (1.09,2.21)**	147	0.86 (0.64,1.14)	39	0.81 (0.47,1.39)
Rh+	170	1	206	1	53	1
Rh-	18	1.57 (0.97,2.56)	10	0.74 (0.39,1.39)	6	1.70 (0.73,3.96)

^a^Cox regression models adjusted for age, smoking, socioeconomic status, ethnicity, residence, education, and opium use.

*P <0.05, **P <0.01. CI, confidence interval; ESCC: esophageal squamous cell carcinoma; HR: hazard ratio; MR: mortality rate per 100,000 person-years.

Absence of the D antigen of Rhesus (Rh) blood group (Rh-) was less common in Turkmen than in non-Turkmen (5.9% versus 8.2%), but there was no association between Rh positivity and any of the study outcomes. We also tested the interaction between Rh group and ABO blood type, and the results were not significant (data not shown). In a subgroup of 11,418, for whom biochemistry test results were available, the association between cardiovascular risk factors and ABO blood groups was assessed. As Table 4 shows, compared with blood group O, group A was associated with higher total cholesterol and low-density lipoprotein (LDL), while group B had lower lipid levels. In total, the only significant difference between non-O and O groups was higher blood glucose in the former (Table 4).

**Table 4:** **Cardiovascular risk factors in a random subgroup (n = 11,418) of Golestan Cohort Study participants according to blood group phenotype**

**Variable**	**Blood group**				
	**O (number = 3,489)**	**A (number = 3,738)**	**B (number = 3,089)**	**AB (number = 1,102)**	**Non-O (number = 7,929)**
**Total cholesterol** ^**a**^	202.9 (43.5)	205.6 (43.8)**	200.5 (41.7)*	201.0 (41.3)	203.0 (42.7)
**HDL**	59.4 (14.8)	59.9 (14.8)	59.2 (14.4)	59.1 (14.5)	59.5 (14.6)
**LDL**	116.2 (36.1)	118.8 (35.4)**	114.2 (34.4)*	115.9 (34.5)	116.6 (35.0)
**Triglycerides**	139.8 (98.3)	139.2 (102.0)	139.3 (98.9)	132.3 (77.9)*	138.3 (97.8)
**FBG**	103.7 (39.5)	105.6 (41.1)	104.8 (40.7)	106.0 (45.3)*	105.4 (41.6)*
**Systolic blood pressure**	125.8 (20.9)	125.7 (20.6)	125.7 (21.0)	124.9 (20.5)	125.6 (20.8)
**Diastolic blood pressure**	77.8 (12.5)	77.4 (12.5)	77.5 (12.6)	77.7 (12.4)	77.5 (12.5)
**BMI**	27.1 (5.3)	27.1 (5.3)	27.1 (5.4)	27.0 (5.3)	27.1 (5.3)
**Waist circumference**	94.6 (13.8)	94.6 (13.8)	94.6 (14.1)	94.2 (14.0)	94.5 (13.9)

^a^Numbers are mean (standard deviation). *P <0.05, **P < 0.01. *P* values adjusted for age, sex, place of residence, education, smoking in pack-years, opium use, and socioeconomic status. Blood lipids and glucose models were also adjusted for BMI. BMI: body mass index; FBG: fasting blood glucose; HDL: high-density lipoproteins; LDL: low-density lipoproteins.

## Discussion

Our results show that individuals with non-O blood groups have higher overall and cardiovascular mortality. They also demonstrate an association between group A and B blood groups and gastric cancer.

The association of non-O blood groups with the incidence of different vascular diseases has been known for some time, although there has been some controversy because of the paucity of information from large prospective studies [17]. In one of the few such studies before the current report, the combined analysis of the Nurses’ Health Study and the Health Professionals Follow-up Study, 6.27% of the coronary heart disease (CHD) cases were attributable to non-O blood groups [7]. However, this study did not report all-cause or cardiovascular mortality. In another study, among 4,901 patients with ischemic heart disease, those with non-O blood groups had higher cardiac mortality [18]. Non-O blood groups are also associated with earlier onset of coronary artery disease [19], more extensive myocardial necrosis and visible thrombus [20]. The two main mechanisms proposed for these associations include the effect of ABO blood groups on serum cholesterol and their influence on hemostasis [8]. Some studies have shown an association between non-O blood groups, particularly group A, and hypercholesterolemia [21], while others have failed to show such an association [22]. The association of variation at the ABO blood group locus with plasma lipid levels has been seen in a GWAS of more than 100,000 individuals of European descent [23]. We also observed higher total cholesterol and LDL levels in people with blood group A, but we think that this alone cannot explain the increased mortality among non-O blood groups mainly because, compared with blood group O, plasma lipid levels were only higher in group A, and these levels were actually lower in other non-O blood types. A recent study also estimated that about 10% of the CHD risk associated with non-O blood groups, is mediated by its influence on LDL cholesterol levels [24].

The association of non-O blood groups with cardiovascular mortality may also be due to the higher levels of vWF and factor VIII in these individuals [8]. A GWAS study found that ABO locus showed the top signal for myocardial infarction in patients with angiographic coronary artery disease (CAD), and concluded that the variation linked to group O and reduced vWF, was protective against myocardial infarction in CAD patients [25]. vWF levels are approximately 25% to 30% higher in people with non-O blood groups [2]. This effect is a direct functional effect and is not due to an association of ABO locus with another gene [26], and the ABH antigenic structures are present on the circulating vWF [2]. Higher levels of vWF have been shown to be independently associated with increased cardiovascular and all-cause-mortality in humans [27], and atherosclerotic plaque progression in mice [28]. The reasons for this association may be the direct role of vWF in platelet adhesion, aggregation and thrombogenesis, although some investigators believe that other mechanisms might be involved as well [29].

Evidence suggests that blood groups A, B and AB probably have a similar effect on the circulating vWF. [30] Blood group A consists of two major subgroups, A1 (about 80%) and A2 (about 20%). In this study, we did not check for differentiation between these subtypes. However, it has been reported that A2 blood group has approximately 47% lower risk of venous thromboembolism compared to other non-O blood groups [31,32]. This lower risk has been suggested to be caused by decreased glycosylation of H antigen, due to a 30- to 50-fold lower glycosyltransferase activity associated with the A2 allele compared to the A1 allele [33]. The impact of such differences on CVD risk and mortality is not clear.

To the best of our knowledge, this is the first study to investigate ABO blood group in relation to cancer mortality, and unlike CVD mortality, we did not observe a significant association for mortality due to all cancers combined. However, incident gastric cancer, which is the second most common cancer in our population, was associated with both blood groups A and B. The association of gastric cancer with blood group A has been observed in many previous studies [10] and is thought to be linked to an altered inflammatory response to *Helicobacter pylori*, particularly cagA positive strains [4]. In the largest study so far, a 35-year follow-up of one million Swedish and Danish blood donors showed an increased risk of gastric cancer among individuals with blood group A compared to group O [34]. However, most previous reports have shown risk estimates of around 1.2 [6], while we observed a stronger association. Our study is also one of the few studies to show an association between blood group B and gastric cancer [11].

One limitation of our study is the left censoring of the mortality data, although most deaths in our population before age 40 (our cohort’s minimum enrollment age) are due to accidents which were not the focus of our evaluation [35]. Also, there were not enough events in the subgroup with available biochemistry data (because of the short follow-up duration in this subgroup), to allow direct analysis of the mediation effect of biochemical changes in the ABO-mortality association.

## Conclusions

We showed that, in apparently healthy individuals, 5.9% of total deaths due to medical causes and 8.9% of cardiovascular deaths were attributable to having non-O blood groups, and these blood groups were also associated with a higher risk of gastric cancer. These findings support the clinical importance of blood group determination in assessing health risks beyond its application in transfusion medicine.

## References

[1] Garratty G (2000). Blood groups and disease: a historical perspective. Transfus Med Rev.

[2] Jenkins PV, O’Donnell JS (2006). ABO blood group determines plasma von Willebrand factor levels: a biologic function after all?. Transfusion.

[3] Cserti CM, Dzik WH (2007). The ABO blood group system and Plasmodium falciparum malaria. Blood.

[4] Sharara AI, Abdul-Baki H, ElHajj I, Kreidieh N, Kfoury Baz EM (2006). Association of gastroduodenal disease phenotype with ABO blood group and Helicobacter pylori virulence-specific serotypes. Dig Liver Dis.

[5] Yamamoto F, Cid E, Yamamoto M, Blancher A (2012). ABO research in the modern era of genomics. Transfus Med Rev.

[6] Liumbruno GM, Franchini M (2013). Beyond immunohaematology: the role of the ABO blood group in human diseases. Blood Transfus.

[7] He M, Wolpin B, Rexrode K, Manson JE, Rimm E, Hu FB, Qi L (2012). ABO blood group and risk of coronary heart disease in two prospective cohort studies. Arterioscler Thromb Vasc Biol.

[8] Franchini M, Mannucci PM: **ABO blood group and thrombotic vascular disease.** *Thromb Haemost* 2014, in press. 10.1160/TH14-05-0457 25187297

[9] Iodice S, Maisonneuve P, Botteri E, Sandri MT, Lowenfels AB (2010). ABO blood group and cancer. Eur J Cancer.

[10] Liumbruno GM, Franchini M (2014). Hemostasis, cancer, and ABO blood group: the most recent evidence of association. J Thromb Thrombolysis.

[11] Nakao M, Matsuo K, Ito H, Shitara K, Hosono S, Watanabe M, Ito S, Sawaki A, Iida S, Sato S, Yatabe Y, Yamao K, Ueda R, Tajima K, Hamajima N, Tanaka H (2011). ABO genotype and the risk of gastric cancer, atrophic gastritis, and Helicobacter pylori infection. Cancer Epidemiol Biomarkers Prev.

[12] Pourshams A, Khademi H, Malekshah AF, Islami F, Nouraei M, Sadjadi AR, Jafari E, Rakhshani N, Salahi R, Semnani S, Kamangar F, Abnet CC, Ponder B, Day N, Dawsey SM, Boffetta P, Malekzadeh R (2010). Cohort profile: The Golestan Cohort Study–a prospective study of oesophageal cancer in northern Iran. Int J Epidemiol.

[13] Khademi H, Etemadi A, Kamangar F, Nouraie M, Shakeri R, Abaie B, Pourshams A, Bagheri M, Hooshyar A, Islami F, Abnet CC, Pharoah P, Brennan P, Boffetta P, Dawsey SM, Malekzadeh R (2010). Verbal autopsy: reliability and validity estimates for causes of death in the Golestan Cohort Study in Iran. PLoS One.

[14] Islami F, Kamangar F, Nasrollahzadeh D, Aghcheli K, Sotoudeh M, Abedi-Ardekani B, Merat S, Nasseri-Moghaddam S, Semnani S, Sepehr A, Wakefield J, Møller H, Abnet CC, Dawsey SM, Boffetta P, Malekzadeh R (2009). Socio-economic status and oesophageal cancer: results from a population-based case–control study in a high-risk area. Int J Epidemiol.

[15] Khademi H, Malekzadeh R, Pourshams A, Jafari E, Salahi R, Semnani S, Abaie B, Islami F, Nasseri-Moghaddam S, Etemadi A, Byrnes G, Abnet CC, Dawsey SM, Day NE, Pharoah PD, Boffetta P, Brennan P, Kamangar F (2012). Opium use and mortality in Golestan Cohort Study: prospective cohort study of 50,000 adults in Iran. BMJ.

[16] Hanley JA (2001). A heuristic approach to the formulas for population attributable fraction. J Epidemiol Community Health.

[17] Wu O, Bayoumi N, Vickers MA, Clark P (2008). ABO(H) blood groups and vascular disease: a systematic review and meta-analysis. J Thromb Haemost.

[18] Carpeggiani C, Coceani M, Landi P, Michelassi C, L’Abbate A (2010). ABO blood group alleles: a risk factor for coronary artery disease. An angiographic study. Atherosclerosis.

[19] Cesena FH, da Luz PL (2006). ABO blood group and precocity of coronary artery disease. Thromb Res.

[20] Ketch TR, Turner SJ, Sacrinty MT, Lingle KC, Applegate RJ, Kutcher MA, Sane DC (2008). ABO blood types: influence on infarct size, procedural characteristics and prognosis. Thromb Res.

[21] Garrison RJ, Havlik RJ, Harris RB, Feinleib M, Kannel WB, Padgett SJ (1976). ABO blood group and cardiovacular disease: the Framingham study. Atherosclerosis.

[22] Amirzadegan A, Salarifar M, Sadeghian S, Davoodi G, Darabian C, Goodarzynejad H (2006). Correlation between ABO blood groups, major risk factors, and coronary artery disease. Int J Cardiol.

[23] Teslovich TM, Musunuru K, Smith AV, Edmondson AC, Stylianou IM, Koseki M, Pirruccello JP, Ripatti S, Chasman DI, Willer CJ, Johansen CT, Fouchier SW, Isaacs A, Peloso GM, Barbalic M, Ricketts SL, Bis JC, Aulchenko YS, Thorleifsson G, Feitosa MF, Chambers J, Orho-Melander M, Melander O, Johnson T, Li X, Guo X, Li M, Shin Cho Y, Jin Go M, Jin Kim Y (2010). Biological, clinical and population relevance of 95 loci for blood lipids. Nature.

[24] Chen Y, Chen C, Ke X, Xiong L, Shi Y, Li J, Tan X, Ye S (2014). Analysis of circulating cholesterol levels as a mediator of an association between ABO blood group and coronary heart disease. Circ Cardiovasc Genet.

[25] Reilly MP, Li M, He J, Ferguson JF, Stylianou IM, Mehta NN, Burnett MS, Devaney JM, Knouff CW, Thompson JR, Horne BD, Stewart AF, Assimes TL, Wild PS, Allayee H, Nitschke PL, Patel RS, Martinelli N, Girelli D, Quyyumi AA, Anderson JL, Erdmann J, Hall AS, Schunkert H, Quertermous T, Blankenberg S, Hazen SL, Roberts R, Myocardial Infarction Genetics Consortium, Wellcome Trust Case Control Consortium (2011). Identification of ADAMTS7 as a novel locus for coronary atherosclerosis and association of ABO with myocardial infarction in the presence of coronary atherosclerosis: two genome-wide association studies. Lancet.

[26] Souto JC, Almasy L, Muniz-Diaz E, Soria JM, Borrell M, Bayen L, Mateo J, Madoz P, Stone W, Blangero J, Fontcuberta J (2000). Functional effects of the ABO locus polymorphism on plasma levels of von Willebrand factor, factor VIII, and activated partial thromboplastin time. Arterioscler Thromb Vasc Biol.

[27] Jager A, van Hinsbergh VW, Kostense PJ, Emeis JJ, Yudkin JS, Nijpels G, Dekker JM, Heine RJ, Bouter LM, Stehouwer CD (1999). Von Willebrand factor, C-reactive protein, and 5-year mortality in diabetic and nondiabetic subjects: the Hoorn Study. Arterioscler Thromb Vasc Biol.

[28] Gandhi C, Ahmad A, Wilson KM, Chauhan AK (2014). ADAMTS13 modulates atherosclerotic plaque progression in mice via a VWF-dependent mechanism. J Thromb Haemost.

[29] van Schie MC, van Loon JE, de Maat MP, Leebeek FW (2011). Genetic determinants of von Willebrand factor levels and activity in relation to the risk of cardiovascular disease: a review. J Thromb Haemost.

[30] Thompson SG, Kienast J, Pyke SD, Haverkate F, van de Loo JC (1995). Hemostatic factors and the risk of myocardial infarction or sudden death in patients with angina pectoris. European Concerted Action on Thrombosis and Disabilities Angina Pectoris Study Group. N Engl J Med.

[31] Tregouet DA, Heath S, Saut N, Biron-Andreani C, Schved JF, Pernod G, Galan P, Drouet L, Zelenika D, Juhan-Vague I, Alessi MC, Tiret L, Lathrop M, Emmerich J, Morange PE (2009). Common susceptibility alleles are unlikely to contribute as strongly as the FV and ABO loci to VTE risk: results from a GWAS approach. Blood.

[32] Heit JA, Armasu SM, Asmann YW, Cunningham JM, Matsumoto ME, Petterson TM, De Andrade M (2012). A genome-wide association study of venous thromboembolism identifies risk variants in chromosomes 1q24.2 and 9q. J Thromb Haemost.

[33] Yamamoto F, McNeill PD, Hakomori S (1992). Human histo-blood group A2 transferase coded by A2 allele, one of the A subtypes, is characterized by a single base deletion in the coding sequence, which results in an additional domain at the carboxyl terminal. Biochem Biophys Res Commun.

[34] Edgren G, Hjalgrim H, Rostgaard K, Norda R, Wikman A, Melbye M, Nyren O (2010). Risk of gastric cancer and peptic ulcers in relation to ABO blood type: a cohort study. Am J Epidemiol.

[35] Forouzanfar MH, Sepanlou SG, Shahraz S, Dicker D, Naghavi P, Pourmalek F, Mokdad A, Lozano R, Vos T, Asadi-Lari M, Sayyari AA, Murray CJ, Naghavi M (2014). Evaluating causes of death and morbidity in Iran, global burden of diseases, injuries, and risk factors study 2010. Arch Iran Med.

